# Biochemical Composition and Biological Activities of Date Palm (*Phoenix dactylifera* L.) Seeds: A Review

**DOI:** 10.3390/biom12111626

**Published:** 2022-11-03

**Authors:** Maryam Abdulraheem Alkhoori, Amanda Shen-Yee Kong, Mariam Nasser Aljaafari, Aisha Abushelaibi, Swee-Hua Erin Lim, Wan-Hee Cheng, Chou-Min Chong, Kok-Song Lai

**Affiliations:** 1 Health Sciences Division, Abu Dhabi Women’s College, Higher Colleges of Technology, Abu Dhabi P.O. Box 41012, United Arab Emirates; 2 School of Biosciences, University of Nottingham Malaysia, Jalan Broga, Semenyih 43500, Malaysia; 3 Office of the Executive Campus Director, Abu Dhabi Colleges, Higher Colleges of Technology, Abu Dhabi P.O. Box 41012, United Arab Emirates; 4 Faculty of Health and Life Sciences, INTI International University, Persiaran Perdana BBN, Putra Nilai, Nilai 71800, Malaysia; 5 Department of Aquaculture, Faculty of Agriculture, Universiti Putra Malaysia, Serdang 43400, Malaysia

**Keywords:** *Phoenix dactylifera*, date seed, antimicrobial, antioxidant, anticancer, antidiabetic, anti-inflammatory

## Abstract

Date palm (*Phoenix dactylifera* L.) is an essential agricultural crop in most Middle Eastern countries, and its fruit, known as dates, is consumed by millions of people. Date seeds, a by-product of the date fruit processing industry, are a waste product used as food for domestic farm animals. Date seeds contain abundant sources of carbohydrates, oil, dietary fiber, and protein; they also contain bioactive phenolic compounds that may possess potential biological properties. In addition, its rich chemical composition makes date seeds suitable for use in food product formulation, cosmetics, and medicinal supplements. This review aims to provide a discourse on the nutritional value of date seeds. The latest data on the cytotoxicity of date seed compounds against cancer cell lines, its ability to combat diabetes, antioxidant potential, antimicrobial effect, and anti-inflammatory activity will be provided, considering its potential to be a nutritional therapeutic agent for chronic diseases. Application of date seeds in the form of powder and oil will also be discussed.

## 1. Introduction

Date palm (*Phoenix dactylifera* L.) is widely cultivated in tropical and subtropical regions, notably in the Middle East and North Africa [1]. Globally, the gross production value of date fruits has been rising considerably since the 20th century, with the highest value seen at more than US $14 billion in 2020 [2]. Date palm trees and fruits drive the economic and social aspects of date-producing countries [3,4]. During the holy month of Ramadan, since the era of Prophet Mohammed (Peace Be Upon Him), Muslims popularly break their fasts by eating dates due to the nutritional and medicinal values mentioned in the Holy Quran sections [5].

Date seeds, a by-product of date fruit production, are usually treated as waste, utilized as animal feed or just disposed of [1]. However, date seeds comprise 10–15% of date fruit fresh weight, and are rich in phenolic compounds [6]. There have been extensive studies carried out on the effects of date seeds in terms of pharmacological activities, such as antioxidant, anti-inflammation, antidiabetic, antibacterial, and antiviral properties [7,8]. Owing to its health benefits, date seeds possess high potential as a nutritional therapeutic agent for several chronic diseases [9]. This paper reviews the date seed nutritional components, biological activities, and its application in medicine and other non-conventional uses.

## 2. Nutritional Components

### 2.1. Amino Acids

The amino acid composition in date seeds is variable depending on the stage of date seed maturation. The major amino acids contained in the date seed are glutamic acid (16.44 g/100 g), phenylalanine (5.93 g/100 g), and leucine (6.10 g/100 g) [10]. Meanwhile, Shina and colleagues (2013) reported that aspartic acid (1.72 g/100 g), alanine (1.2 g/100 g), and tyrosine (1.2 g/100 g) are the major non-essential amino acids, while leucine (1.7 g/100 g), lysine (1.1 g/100 g), and phenylalanine (1.08 g/100 g) are the major essential amino acids, following implementation of an automatic amino acid analyzer [11]. Furthermore, non-proteogenic amino acids can bind with antibodies to produce T lymphocytes, eliminate toxic compounds in the liver, and reduce the creatinine levels in the body, including g-amino-n-butyric acid and 1-aminocyclopropane-1- carboxylic acid [12,13].

### 2.2. Dietary Fibers

Studies have found that dates contain more dietary fibers than cereals [14]. Functional dietary fibers can be found in date seeds, such as tannin, lignin, pectin, and hemicellulose components. Attia and colleagues (2021) reported a higher fiber content in date seeds than in yellow corn and barley [15]. Date seeds contain fiber fractions at a range of 39.6% to 57.5% for acid detergent fiber (cellulose + lignin), 51.6% to 75.0% for neutral detergent fiber (hemicellulose + cellulose + lignin), 12.0% to 17.5% for hemicellulose, 26.1% to 42.5% for cellulose, and 7.21% to 11.0% for lignin.

### 2.3. Minerals and Vitamins

Techniques such as gas chromatography and high-performance liquid chromatography (HPLC) have shown that date seeds contain vitamins consisting of vitamin A, vitamin B1 (thiamine), vitamin B2 (riboflavin), vitamin C, vitamin E, vitamin K, folate, and different types of minerals, with the most abundant being potassium [16,17]. Other minerals include boron, calcium, cobalt, copper, iron, magnesium, phosphorus, fluorine, selenium, sodium, and zinc [18]. Of these, potassium, phosphorus, and iron are present in higher concentrations among the minerals. Owing to their low sodium ratio level, consumption of date seeds is recommended for people with hypertension. Hinkaew and colleagues (2021) reported higher angiotensin-converting enzyme inhibitory activity in date seeds from cell culture origin compared to their flesh. Overall, date seeds exhibited 58.69–61.36% inhibition towards the key enzyme in regulating hypertension. In addition, date seeds also have high fluorine content and can help prevent tooth decay [19]. 

Date oil extracted from its seed is rich in amino acids and vitamins which can significantly promote hair growth [20]. Minerals extracted from date seed oils can generate energy and restore cellular function and growth of Riboflavin [21]. A study in the United Arab Emirates (UAE) has experimented on 18 types of date seed oil, including Khalas, Lulu, Fard, Raziz, and Sokkery, with several vitamins significantly observed through ultra-performance liquid chromatography analysis [22]. It was found that most date seed oils contain high α-tocopherol, followed by α-tocopheryl acetate, γ-tocopherol, and, lastly, Vitamin K1.

### 2.4. Sugars

The soluble sugars in date seeds contain 3.5 g/kg of glucose, 3.8 g/kg of fructose, 3.7 g/kg of stachyose, 3.5 g/kg of sucrose, and 2.2 g/kg of galactose, as reported through UV-visible spectrophotometry [23]. Date seed sugar can control blood sugar levels in diabetic patients with its beneficial powder of insulin-producing properties [24]. El Fouhil and colleagues (2013) reported the potential hypoglycemic efficacy of date seeds by observing an increased level of serum c-peptide in date seed extract (DSE)-insulin-treated diabetic rats in comparison to only insulin-treated diabetic rats [25]. This suggested an increase in endogenous insulin secretion, as confirmed by a compensatory beta-cell hypertrophy seen through immunohistochemistry analysis.

### 2.5. Fatty Acids

Oils extracted from date seeds contain five major fatty acids—oleic, lauric, myristic, linoleic, and palmitic [26]. Through gas chromatography, Biglar and colleagues (2012) reported oleic–lauric oil as the major fatty acid with 33.38% to 51.40% content, followed by lauric acid, with 18.78% to 31.61% content. Myristic acid was reported as the third major fatty acid, followed by palmitic and linoleic acid.

Attia and colleagues (2021) highlighted the importance of date seed oil as a natural source of medium-chain fatty acids and its ability to enhance poultry growth and immunity level [15]. Fatty acid fractions of date seeds were reported in a range of 12.20% to 23.06% for lauric, 9.70% to 11.30% for myristic, 10.11% to 12.70% for palmitic, 1.56% to 3.56% for stearic, 35.1% to 45.80% for oleic, 8.10% to 11.00% for linoleic, and 0.37% to 0.80% for linolenic acids. These values were similar to those from corn, except for myristic and oleic fatty acids which were higher than in corn.

### 2.6. Phenolic Compounds

Date seeds have been found to contain high levels of total polyphenols in comparison to other fruit, such as grapes, nut seeds, and even their flesh [27]. Seven different phenolic acids—including caffeic acid, chlorogenic acid, p-coumaric acid, ferulic acid, gallic acid, syringic acid, and vanillic acid—have been found to exist in date seeds, with the highest content ranging between 109.87 and 141.72 mg/100 g and seen in p-coumaric acid [28]. In addition, analysis of HPLC with a diode-array detector (DAD) data has suggested that rutin represents the most abundant flavonoid in date seeds, with a range of 71.74 to 86.32 mg/100 g, followed by quercetin (23.71–34.06 mg/100 g), and luteolin (9.17–13.24 mg/100 g). Table 1 summarizes the nutritional components of date seeds appraised in this review.

## 3. Biological Activities of Date Seeds

Based on the different nutritional values present in date seeds, numerous studies have looked into their biological activities which might be useful for human health. These activities include antimicrobial, antioxidant, anticancer, antidiabetic, and anti-inflammatory, which will be discussed in the following chapter.

### 3.1. Antimicrobial Activities

The date seed possesses an antimicrobial feature, being able to invade the cytoplasmic membrane of the bacteria [23]. Alrajhi and colleagues (2019) evaluated the antibacterial activity of date seed powder (DSP) extracted using ethyl acetate and hexane [31]. It was found that methicillin-resistant *Staphylococcus aureus* (MRSA) has the highest sensitivity, with an inhibition zone of 20 mm diameter, and was more effective than the common antibiotic gentamycin, which showed only a diameter of 5 mm inhibition. Among the tested gram-negative bacteria, only *Proteus mirabilis* showed significant inhibition towards DSEs.

Anwar and colleagues (2022) recently reported the antimicrobial activities of methanol Ajwa DSEs against gram-negative and gram-positive bacteria [32]. Notable inhibition zones by 100 mg/mL of Ajwa date seed against *S. aureus*, *Escherichia coli*, *Klebsiella pneumonia*, *Pseudomonas aeruginosa*, and *Enterococcus faecalis* were observed at 19 mm, 15 mm, 16 mm, 14 mm, and 16 mm, respectively.

Jassim and colleagues (2010) found that the antiviral activity of DSEs against lytic *Pseudomonas* phage ATCC 14209-B1 led to inhibition of the infectivity of *Pseudomonas* phage ATCC 14209-B1 to *P. aeruginosa*, reduction of the phage function, and disruption of the bacterial lysis [33].

### 3.2. Antioxidant Activities

Anwar and colleagues (2022) recently found a strong antioxidant activity in Ajwa date seed methanol extracts comparable to that of ascorbic acid [32]. In addition, research has also been conducted to explore the beneficial antioxidant effects of date seeds based on their rich source of phenolic compounds [34]. Salomón-Torres and colleagues (2019) found that the polyphenolic content in the seed of ‘Medjool’ dates is ten times higher than that in the pulp [35]. Presence of phenolic compounds such as tannins, flavonoids, saponins, anthraquinones, and coumarins agreed with the Shinoda assay contributed to the antioxidant properties of date seeds, resulting in a lower IC_50_ of 0.0046 g/L by employing 2,2-diphenyl-1-picrylhydrazyl (DPPH) free radical method. Dietary polyphenolics from date seeds may supply substantial antioxidants, promoting health and preventing diseases. 

Bentrad and colleagues (2020) found an antioxidant property of date seeds extracted from the Bent Kbala cultivar using the DPPH method [36]. DSEs demonstrated the highest free radical inhibition rate (89.89 ± 0.02%) and antioxidant activity (IC_50_ = 0.031 μg/mL) compared to other natural antioxidants. These antioxidant properties are associated with phenolic compounds, such as phenolic acids, catechin tannins, and flavonoids contained in the date seeds, and their ability to donate hydrogen to DPPH radicals effectively.

Hilary and colleagues (2021) investigated the total polyphenol content of DSE, DSP, and date seed Arabic pita bread (DSB). It was found that the polyphenol content increased following digestion of DSB [9]. Polyphenols protect cell constituents such as DNA, protein, and lipid against oxidative damage by eliminating free radicals. The DPPH assay and Trolox Equivalent Antioxidant Capacity (TEAC) assay reported an enhanced antioxidant power in DSB after gastric digestion. However, the Ferric Reducing Antioxidant Power (FRAP) assay and the TEAC assay recorded a reduction in the antioxidant activity of DSE at the end of intestinal digestion. The DPPH assay remained unchanged despite the decrease in total polyphenol content. In the case of DSP, only the FRAP assay revealed a reduction in the antioxidant power as the digestion progressed from the gastric to intestinal phase.

In contrast, Platat and colleagues (2019) reported a significant increase in antioxidant effect in human subjects with a single dose of DSE, DSP, and DSB [37]. After 8 and 24 h of ingestion of date seed products, protection against protein and lipid peroxidation was increased. Presence of metabolites such as hydroxybenzoic acids, vanillic acids, protocatechuic acids, and vanillic acid sulphate in the excreted urine validates the role of date seeds in antioxidant activity.

### 3.3. Anticancer Activities

Phytotherapeutic agents such as plant-based cancer treatment are the preferable option, since they are natural, readily available, easily assimilated into the body, and have fewer side effects and lower toxicity [38]. As demonstrated in experimental studies, date seeds can be used as an anticancer treatment since they can successfully restrain malignancies. Hilary and colleagues (2021) revealed the potential of DSEs in reducing the viability of cancer cell lines at high concentrations (1000 μg/mL or above) after long hours of exposure [9]. After 48 h of treatment, the anticancer effect of reduced viability was found in the triple-negative breast cancer cell line (MDA-MB-231), breast cancer cell line (MCF-7), and colon adenocarcinoma cell line (Caco-2). For liver cancer cell line (HepG2) and prostate adenocarcinoma cell line (PC-3), a significant reduction in cell viability was observed with ≥1000 μg/mL of DSE treatment after 24 h of treatment.

Khan and colleagues (2022) reported that *P. dactylifera* seed extracts played a role in inducing cellular apoptosis through intrinsic pathways in the MDA-MB-231 cell line, MCF-7 cell line, and HepG2 cell line [38]. After 24 h of treatment, the cancer cells displayed morphological changes such as non-adherent, detached, and rounded shapes. Apoptotic features were observed after 48 h of exposure. The time- and dose-dependent manner of DSEs were shown by cell viability tests with an observation of a significant reduction in cancer cell growth. This result is suggested to link with the synergistic effect of bioactive agents—rutin and quercetin—present in date seeds. The morphology variation and cell viability remained unchanged in the normal Vero cell line following 24 h and 48 h of exposure. To the best of our knowledge, there is scarce literature available concerning the in vivo experiments for the anticancer properties of date seeds. Hence, it is recommended for future researchers to investigate this issue.

### 3.4. Antidiabetic Activities

Date seed active compounds, such as polyphenols and phenolics, aid in antidiabetic activity by scavenging free radicals, repairing pancreatic cells, reducing lipid peroxidation, and inhibiting enzymes on intestinal glucose absorption [39]. Shakoor and colleagues (2020) found that DSEs regulate glucose homeostasis with its high phenolics and flavanols content [40]. DSEs inhibited two digestive enzymes, pancreatic α-amylase and intestinal α-glucosidase, which are responsible for starch digestion and modulation of the glucose uptake by HepG2. At 400 μg/mL and 900 μg/mL of DSE, maximum enzyme inhibition was observed in α-amylase and α-glucosidase. Increased glucose uptake by HepG2 cells was observed at 40 μg/mL and 100 μg/mL of DSE compared to the control without insulin.

Hilary and colleagues (2021) investigated the metabolic changes in the preadipocyte (3T3-L1) and myoblast cells (C2C12) with DSE treatment and found enhanced glucose uptake in 3T3-L1 cells [9]. 3T3-L1 cells reported increased glucose uptake with 200 μg/mL of DSE treatment combined with insulin. The presence of polyphenols in date seeds plays a crucial role in enhancing the insulin signaling pathway. C2C12 muscle cells showed an improved glucose uptake with DSE treatment, but the results were inconsistent with different replicates.

Abdelaziz and colleagues (2015) revealed the potential of aqueous suspension of *P. dactylifera* seeds (aqPDS) in protecting streptozotocin (STZ)-induced diabetic rats from early diabetic complications in both liver and kidney [41]. Significant reduction of blood glucose level by 51% was observed in groups subjected to aqPDS treatment compared to the untreated diabetic group, but the normal blood glucose values were not restored. Four weeks of continuous aqPDS administration in STZ-induced diabetic rats not only decreased the levels of total cholesterol, triglycerides, serum urea, and creatinine, but also restored AST and ALT levels to normal. This indicated that intake of *P. dactylifera* seeds could lessen the renal damage induced by diabetes. 

### 3.5. Anti-Inflammatory Activities

Oxidative stress such as protein peroxidation, lipid peroxidation, and DNA mutagenesis induces overproduction of reactive oxygen species and activates inflammatory mediators involved in the development and progression of chronic diseases, including cardiovascular diseases [42,43]. Saryono and colleagues (2020) carried out a study to investigate the anti-inflammatory mechanism of date seeds in middle-aged women. They found a significant reduction in pro-inflammatory cytokines and cyclooxygenase (COX) expression [8]. The level of interleukin-1β, transforming growth factor-β, COX-1, and COX-2 decreased upon uptake of date seeds treatment in middle-aged female subjects. 

**Figure 1:**
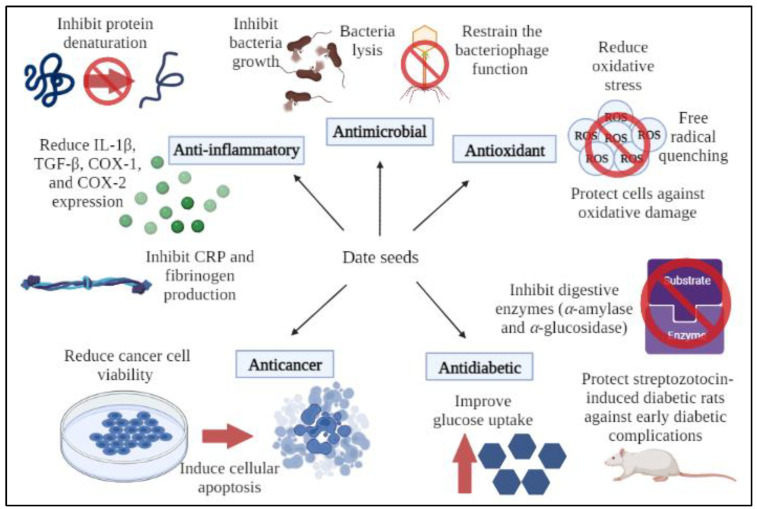
A schematic representation of the biological activities of date seeds. The highly valuable constituents of date seeds contribute to its antimicrobial, antioxidant, anticancer, antidiabetic, and anti-inflammatory properties.

Bouhlali and colleagues (2020) revealed the anti-inflammatory properties of DSE by inhibiting protein denaturation, stabilising the lysosomal membranes, scavenging nitric oxide free radicals, and inhibiting C-reactive protein and fibrinogen production [44]. It is suggested that these mechanisms may be linked to the presence of phenolic compounds such as quercetin, rutin, caffeic acids, p-coumaric, and gallic acids in the date seeds, as observed through HPLC-DAD analysis. Among the four varieties of date seeds (Boufgous, Bousthammi, Jihl, and Majhoul), Jihl cultivar represents the most effective date seed by showing the strongest anti-inflammatory ability. Table 2 summarizes the biological activities of date seeds, while Figure 1 shows the schematic representation of biological activities of date seeds appraised in this review.

## 4. Industrial Value of Date Seeds in a Commercial Application

DSP has been used in traditional remedies and for synthesizing citric acid and protein by *Candida lipolytica*, *Aspergillus oryzae*, and *Candida utilis* [19]. The date seed oil contains saturated and unsaturated fatty acids such as lauric and oleic acid [4]. Mrabet and colleagues (2020) reported the benefits of date seed oils for cooking, frying, or seasoning as a result of its excellent stability against oxidative rancidity and high temperature. Date seed oils were also used to replace conventional corn oil for mayonnaise production, and the mayonnaise outcome has better sensory characteristics [45].

Owing to its versatile antioxidant activities, date seeds can protect human skin against UV-B and UV-A radiation which can damage the skin [4]. Bioactive constituents such as flavonoids, phenolics, and phenolic acids inhibit free radicals production that causes cellular damage to the skin [17]. Lecheb and colleagues (2015) proposed a cosmetic cream using aqueous extract and oil from date seeds [46]. The spreadability, viscosity, and rheological behavior of the formulated cream were similar to those of the commercial cosmetic cream. Phytohormones in date seed oil possess anti-aging properties, reducing wrinkles and improving skin elasticity [47]. In recent years, date seed oil has been used to formulate body creams, soaps, hair products, and sunscreens for daily usage. The date seed oil contains vitamins, carotenes, and other nutrients that help maintain a healthy scalp, promote normal hair growth, and support the nutritional functions of hair follicles [20].

Moreover, date seed oil can be used as biodiesel owing to its low content of free fatty acids [4]. Using date seeds as a feedstock would bring enormous economic benefits and potential for future industrial operations [48].

## 5. Future Prospect

Long-term exposure to high oxidative stress plays a crucial role in the development of various chronic non-communicable diseases [49]. The ability of date seeds to scavenge free radicals and protect cells from oxidative damage might be the key mechanism of remedial action toward chronic diseases. In addition, the oil extracted from date seeds contains monounsaturated fatty acids and lipid-soluble antioxidants such as phenols and phytosterols that can reduce the risk of multiple diseases. With an abundance of date seeds, the oil extracted from the seeds might be useful to provide a chance for future enterprises in areas such as human foods application, medicine, biodiesel, and plastic manufacturing. Date seeds would evolve from waste to feedstock to promote economic and social growth in the date fruit production and manufacturing fields [48]. Innovative and optimized technology that improves the recovery and quality of DSEs will make the date seed a valuable source of antioxidants for nutraceuticals and functional food applications [3]. Although date seeds have been consumed over the centuries by humans and animals, controlled toxicity and bioavailability studies are still necessary to gauge the effectiveness of the medications and their potential to elicit a toxic response as the standard for every clinical trial.

## 6. Conclusions

Date seeds contain rich sources of nutritional functional components that may help to combat deficiency-related diseases, infections, and disorders related to oxidative stress. Current pharmacological studies have found that DSEs can regulate human health through several biological activities. However, an in-depth investigation is required to characterize polyphenols and other unique bioactive compounds, including their mechanism, as well as to establish ideal doses without causing harm. The potential of date seeds as a treatment against chronic diseases brings enormous promises and possibilities for future research.

## Figures and Tables

**Table 1:** A summary of the highly extractable and valuable components of data seeds.

Component	Content (%)	References
Protein	2.3–6.4	[29]
Fat	5.0–13.2	[29]
Ash	0.82–1.14	[30]
Carbohydrate	2.43–4.65	[30]
Dietary fiber	22.5–80.2	[29]
Phenolics	5.072	[27]
Magnesium	0.058–0.090	[30]
Calcium	0.014–0.034	[30]
Phosphorus	0.110–0.134	[30]
Potassium	0.175–0.240	[30]
Sodium	0.008–0.013	[30]

**Table 2:** A summary of biological activities of date seeds.

Biological Activities	Date Seed Cultivar	Type and List of Samples	Findings	References
Antimicrobial	Not stated	Bacterial strains: *P. aeruginosa* *E. coli* *K. pneumonia* *P. vulgaris* *P. mirabilis* MRSA *E. faecalis*	The highest sensitivity was observed in MRSA, with an inhibition zone of 20 mm diameter and was more effective than the common antibiotic gentamycin, which showed only a diameter of 5 mm inhibition. Among the tested gram-negative bacteria, only *P. mirabilis* showed significant inhibition towards DSE.	[31]
	Ajwa	Bacterial strains: *S. aureus* ATCC 29213 *E. coli* ATCC 25922 *K. pneumonia* ATCC 700603 *P. aeruginosa* ATCC 27853 *E. faecalis* ATCC 29212	Notable inhibition zone by 100 mg/mL of Ajwa date seed against *S. aureus*, *E. coli*, *K. pneumonia*, *P. aeruginosa*, and *E. faecalis* was observed at 19 mm, 15 mm, 16 mm, 14 mm, and 16 mm, respectively.	[32]
	Not stated	Bacteria strain: *P. aeruginosa* ATCC 25668 Bacteriophage: Lytic *Pseudomonas* phage ATCC 14209-B1	DSEs significantly inhibited infectivity of *Pseudomonas* phage ATCC 14209-B1 to *P. aeruginosa*, reduced phage function, and completely disrupted the bacterial lysis.	[33]
Antioxidant	Ajwa	Not stated	Strong antioxidant nature of Ajwa date seed methanol extracts was comparable to that of ascorbic acid.	[32]
	Medjool	Not stated	Approximately ten times the total polyphenolic content in the seed of ‘Medjool’ dates grown in Mexico compared to the quantity in the pulp. Shinoda assay confirmed presence of phenolic compounds such as tannins, flavonoids, saponins, anthraquinones, and coumarins. By employing DPPH free radical method, date seeds showed a much lower IC_50_ result of 0.0046 g/L.	[35]
	Bent Kbala	Not stated	DSE demonstrated the highest free radical inhibition rate (89.89 ± 0.02%) and the highest antioxidant activity (IC_50_ = 0.031 μg/mL) compared to other natural antioxidants. These antioxidant properties are related to phenolic compounds, such as phenolic acids, catechin tannins, and flavonoids present in the date seeds, and their ability to donate hydrogen to DPPH radicals.	[36]
	Khalas	Not stated	Increased polyphenol content following digestion of DSB. Both the DPPH and TEAC assays demonstrated a significant improvement in antioxidant power in DSB after gastric digestion. FRAP assay and TEAC assay recorded a significant decrease in the antioxidant activity of DSE at the end of intestinal digestion. The DPPH assay showed unchanged results in the antioxidant activity despite the decrease in total polyphenol content. In the case of the DSP, only the FRAP assay revealed a reduction in the antioxidant power as the digestion progressed from the gastric to intestinal phase.	[9]
	Khalas	16 healthy adults (8 women and 8 men) Human serum and plasma Human urine	Significant increase in antioxidant effect with a single dose of DSE, DSP, and DSB. Increased protection against protein and lipid peroxidation was observed up to 8 and 24 h after ingestion of date seed products. Presence of metabolites such as hydroxybenzoic acids, vanillic acids, protocatechuic acids, and vanillic acid sulphate in the excreted urine supports the involvement of date seeds in antioxidant activity.	[37]
Anticancer	Khalas	Cancer cell lines: human breast adenocarcinoma cell lines MCF-7 and MDA-MB-231 human colon adenocarcinoma cell line Caco-2 human hepatocyte carcinoma cell line HepG2 human prostate adenocarcinoma cell line PC-3	DSEs reduced viability of cancer cell lines at high concentrations (1000 μg/mL or above) following long hours of exposure. The anticancer effect of reduced viability was observed in the MDA-MB-231, MCF-7, and Caco-2 after 48 h of treatment. HepG2 and PC-3 showed a significant cell viability reduction with ≥1000 μg/mL of DSE treatment after 24 h.	[9]
	Ajwa	Cancer cell lines: human triple-negative breast cancer cell line MDA-MB-231 human estrogen receptor and progesterone receptor-positive breast cancer cell line MCF-7 human liver carcinoma cell line HepG2 normal kidney epithelial Vero cell lines	*P. dactylifera* seed extracts induced cellular apoptosis through intrinsic pathways in the MDA-MB-231 cell line, MCF-7 cell line, and HepG2 cell line. Treated cancer cells displayed morphological changes such as non-adherent, detached, and rounded shapes after 24 h of exposure. Apoptotic features were observed after 48 h of exposure. The time- and dose-dependent manner of DSEs were shown by cell viability tests with an observation of a significant reduction in cancer cell growth. This cytotoxic activity was attributed to the synergistic effect of bioactive agents—rutin and quercetin—present in date seeds. No changes in morphology variation and cell viability were observed in the normal Vero cell line following 24 h and 48 h of exposure.	[38]
Antidiabetic	Khalas	HepG2 liver cells culture	DSEs inhibited two digestive enzymes, pancreatic α-amylase and intestinal α-glucosidase, which are responsible for starch digestion and modulation of the glucose uptake by HepG2. Maximum enzyme inhibition was observed in α-amylase and α-glucosidase at 400 μg/mL and 900 μg/mL of DSE, respectively. Increased glucose uptake by HepG2 cells was observed with doses of DSEs at 40 μg/mL and 100 μg/mL compared to the control without insulin.	[40]
	Khalas	C2C12 myoblast cell lines, 3T3-L1 preadipocytes	The increase in glucose uptake was observed in 3T3-L1 cells with 200 μg/mL of DSE treatment in combination with insulin. Presence of polyphenols in date seeds is crucial for enhancing the insulin signaling pathway. An improved glucose uptake with DSE treatment was observed in C2C12 muscle cells, but the results were insignificant due to the high variability in the replicates.	[9]
	Hayani	21 Adult male Wistar rats: Group I (Control group): 7 rats received a single intraperitoneal injection of citrate buffer vehicle. Group II (Untreated diabetic group): 7 rats received a single intraperitoneal injection of STZ (50 mg/kg). Group III (aqPDS-treated group): 7 rats received a single intraperitoneal injection of STZ (50 mg/kg) and daily administration of aqPDS (1 g/kg/d) by oral gavage for 4 weeks.	Potential of aqPDS in protecting STZ-induced diabetic rats from early diabetic complications in both liver and kidney. Significant reduction of blood glucose level by 51% was observed in groups subjected to aqPDS treatment compared to the untreated diabetic group, but the normal blood glucose values were not restored. Four weeks of continuous aqPDS administration in STZ-induced diabetic rats not only decreased the levels of total cholesterol, triglycerides, serum urea, and creatinine, but also restored AST and ALT levels to normal. This indicated that intake of *P. dactylifera* seeds could lessen the renal damage induced by diabetes.	[41]
Anti-inflammatory	Deglet Nour	Blood samples of 30 middle-aged women	Significant reductions of pro-inflammatory cytokines and cyclooxygenase (COX) expression were observed. The level of interleukin-1β, transforming growth factor-β, COX-1, and COX-2 decreased following uptake of date seeds treatment in middle-aged female subjects.	[8]
	Boufgous, Bousthammi, Jihl, and Majhoul	36 Wistar strain rats and 30 Swiss abino mice	DSE inhibited protein denaturation, stabilised the lysosomal membranes, showed nitric oxide free radicals scavenging ability, and inhibited C-reactive protein and fibrinogen production. It is suggested that these mechanisms may be linked to the presence of phenolic compounds, such as quercetin, rutin, caffeic acids, p-coumaric, and gallic acids, in the date seeds, as observed through HPLC-DAD analysis. Among the four varieties of date seeds (Boufgous, Bousthammi, Jihl, and Majhoul), Jihl cultivar represents the most effective date seed by showing the strongest anti-inflammatory ability.	[44]
